# The Serum Immunoglobulin E Level: Is There a Relationship With the Clinical Course of the Gianotti-Crosti Syndrome?

**DOI:** 10.3389/fped.2021.643341

**Published:** 2021-02-25

**Authors:** Andrea Bassi, Fausto Pedaci, Teresa Oranges, Chiara Azzari, Luisa Galli, Silvia Ricci, Cesare Filippeschi, Elisabetta Venturini

**Affiliations:** Department of Health Sciences, Anna Meyer Children University Hospital, University of Florence, Florence, Italy

**Keywords:** Gianotti-Crosti syndrome, immunoglobulin, immunoglobulin E–blood, children, itch

## Abstract

**Background:** Gianotti Crosti syndrome (GCS) is a self-healing condition with a spontaneous resolution in 2–6 weeks but, even if rarely, recurrent episodes have been reported.

**Objective:** The aim of this observational study is to investigate serum Immunoglobulin E (IgE) level in children with GCS, evaluating if there is a relationship between IgE level and clinical course of the disease.

**Methods:** Children with GCS diagnosed at a tertiary care children's university hospital between June 2018 and November 2019 were prospectively enrolled. Demographic, clinical and hematochemical data of children investigated were collected. In particular, IgE level were investigated at symptoms onset and, if available, at the following blood tests. Patients were divided in 2 groups on the bases of the clinical course: children with a chronic relapsing course and children who did not present any relapse.

**Results:** Among 29 patients enrolled in this study, 14 (48.3%) children had a chronic relapsing course and 15 (51.7%) did not present any relapse. A statistically significant difference was present considering the length of the disease: 210 days (IQR: 161.25–255) for patients with a chronic relapsing course compared to 40 days (IQR: 30–75) for the other group (*p* < 0.0001). About the median IgE level in the 2 groups, a value about 10 time higher was found in children with chronic course compared to the other group (1,144 vs. 116 U/mL) with a statistically significant difference (*p* < 0.0001).

**Conclusion:** Despite the study limitations, a significant correlation between higher IgE levels and chronic-relapsing course of the GCS can be assumed.

## Introduction

Gianotti-Crosti syndrome (GCS), or infantile papular acrodermatitis, is a distinctive childhood exanthema characterized by papular or papulovescicular lesions that are most prominent on the extremities, buttocks and face (1, 2). Although often asymptomatic, the lesions may be mildly to moderately pruritic. It is known or suspected to be associated with many viral infections, such as Hepatitis B virus, Cytomegalovirus, Coxsackie, Adenovirus, Influenza and, more frequently, Epstein-Barr virus (3, 4). Recent immunizations are often described in association to GCS (5, 6). Although it has been rarely described in adults, it mainly affects children younger than 4 years. The diagnosis is clinical and usually no specific blood tests are required.

It is usually a self-healing condition with a spontaneous resolution in 2–6 weeks, but sometimes it may last several months and recurrent episodes have been reported. No specific treatment for GCS is required, except mild topical steroid cream and oral antihistaminic which may be prescribed for the itch. Recurrence of GCS has been reported in literature (1, 7). According to previous reports, GCS may be more common in individuals with personal or familiar history of atopy (8, 9). GCS has also been reported in association with pediatric idiopathic hypereosinophilic syndrome (10).

The aim of this observational study is to investigate serum Immunoglobulin E (IgE) level in children with GCS and its relationship between the IgE level and the clinical course of the disease. Moreover, IgE level within the first month of disease would be assessed in order to establish a possible predictive role on the length of GCS.

## Methods

### Study Population

All the children with a diagnosis of GCS at the Anna Meyer tertiary care children's university hospital were prospectively enrolled between June 2018 and November 2019. Demographic, clinical and hematochemical data were collected. In particular, the following data were included in the study database: children's sex and age, date of symptoms onset, clinical prodromes before GCS onset, personal history of atopy, disease length, and blood tests result. IgE level were investigated at the first hospital evaluation and, if available, at the following blood tests during follow-up visits.

According to local ethical review board instructions, on hospital admission all patients signed an informed consent for the inclusion in observational studies, with anonymized data extraction.

### Case Definition

GCS was clinically diagnosed through validated diagnostic criteria for GCS proposed by Chuh et al. (11), Chuh (12). In particular these authors differentiated between the positive and negative clinical features for the diagnosis. The positive ones include the presence of monomorphous, flat-topped, pink-brown papules or papulovesicles 1–10 mm in diameter with at least three of the following four sites involved: (I) cheeks, (II) buttocks, (III) extensor surfaces of forearms, and (IV) extensor surfaces of legs, symmetrically distribution and lasting for at least 10 days. The chronically relapsing course has been defined as the appearance of two or more close episodes of the disease. In particular, a chronic relapsing course was considered as a fluctuating course with wax and wane of the lesions within the same episode. Clinical relapses were always double-blind evaluated by two dermatologists confirming the diagnosis of GCS. Negative clinical features were the presence of extensive truncal lesions or scaly lesions (11, 12). These diagnostic criteria were used in our study to identify eligible children.

### Statistical Analysis

Data were recorded in the study database and analyzed by SPSS Statistics, 24.0 statistics software. Categorical variables and frequencies were compared by means of χ^2^ test or Fisher test, as appropriate. Quantitative variables were reported as median and interquartile ranges (IQRs) and they were compared by means of nonparametric tests (Mann–Whitney *U*). *P* < 0.05 was considered statistically significant. Pearson's correlation coefficient (r) has been calculated to see the correlation between IgE levels and time of the blood tests. To assess the diagnostic accuracy of serum IgE levels to predict the chronic relapsing course risk of GCS, we calculated sensitivity, specificity, and the area under the receiver operating characteristic (ROC) curve (AUC).

## Results

A total of 29 patients with GCS (51.7% females) were enrolled during the study period. The median age at onset was 20 months (IQR: 13–29) with a median length of disease of 90 days (IQR: 40–210). Infective prodromes were present in almost half of the patients (*n* = 12, 44.8%), in particular mild to moderate fever, cough, diarrhea, and vomiting or influenza-like symptoms. One patient reported the onset of the lesions 5 days after the immunization for rotavirus.

Patients were divided in 2 groups on the bases of the clinical course: 14 (48.3%) children had a chronic relapsing course and 15 (51.7%) did not present any relapse, as shown in Table 1A. No significant difference in sex distribution, age at onset and clinical prodromes were observed among the 2 groups (*p* = 0.096, *p* = 0.813, and *p* = 0.362, respectively). On the contrary, a statistically significant difference was present considering the length of the disease: 210 days (IQR: 161.25–255) for patients with a chronic relapsing course compared to 40 days (IQR: 30–75) for the other group (*p* < 0.0001). The median time between symptoms onset and the first IgE detection was 17 days (IQR: 3.5–50). For children with a chronically relapsing course this interval was in median of 30 days (IQR: 11–98.75), compared to the other group of 10 days (IQR: 1–20) (*p* < 0.037). Children with a chronically relapsing course displayed also extensive and more itchy lesions than the other group.

**Table 1:** **(A)** Population characteristics considering the disease course. **(B)** IgE level (median, IQR) considering the timing at detection and the disease course.

**A**		**All** ***n*:29**	**Chronically** **relapsing course** ***n*:14(48.3%)**	**Non-chronically relapsing course** ***n*:15 (51.7%)**	***P***
Sex					0.096
Male		51.7%	9/14 (64.3%)	5/15 (33.3%)	
Female		48.3%	5/14 (35.7%)	10/15 (66.7%)	
Age					0.813
Median (months)		20	18	23	
IQR range (months)		(13–29)	(12.75–29)	(14–30)	
Length of disease					**<0.0001**
Median (months)		90	210	40	
IQR range (months)		(40–210)	(161.25–255)	(30–75)	
Clinical prodromes					0.362
Yes		12 (44.8%)	7/13 (50%)	5/16 (33.3%)	
No		17 (55.2%)	7/13 (50%)	10/16 (66.7%)	
**B**	**Time of IgE** **detection**	**–**	**Chronically** **relapsing course** ***n*****:14(48.3%)**	**Non-chronically relapsing course** ***n*****:15 (51.7%)**	***P***
	IgE < 30 days	**–**	1,144.5 (361–4,087.5)	93 (39.5–259)	**0.003**
	IgE 31–90 days	–	531 (371–4,074)	132.5 (78.25–631.3)	0.054
	IgE 91–180 days	–	1,005.5 (760.7–4854.3)		
	IgE > 181 days	–	1,577.5 (1,142–2,412.5)		

*The bold values are reported to highlighted the significative difference respect to the other variables*.

**Figure 1:**
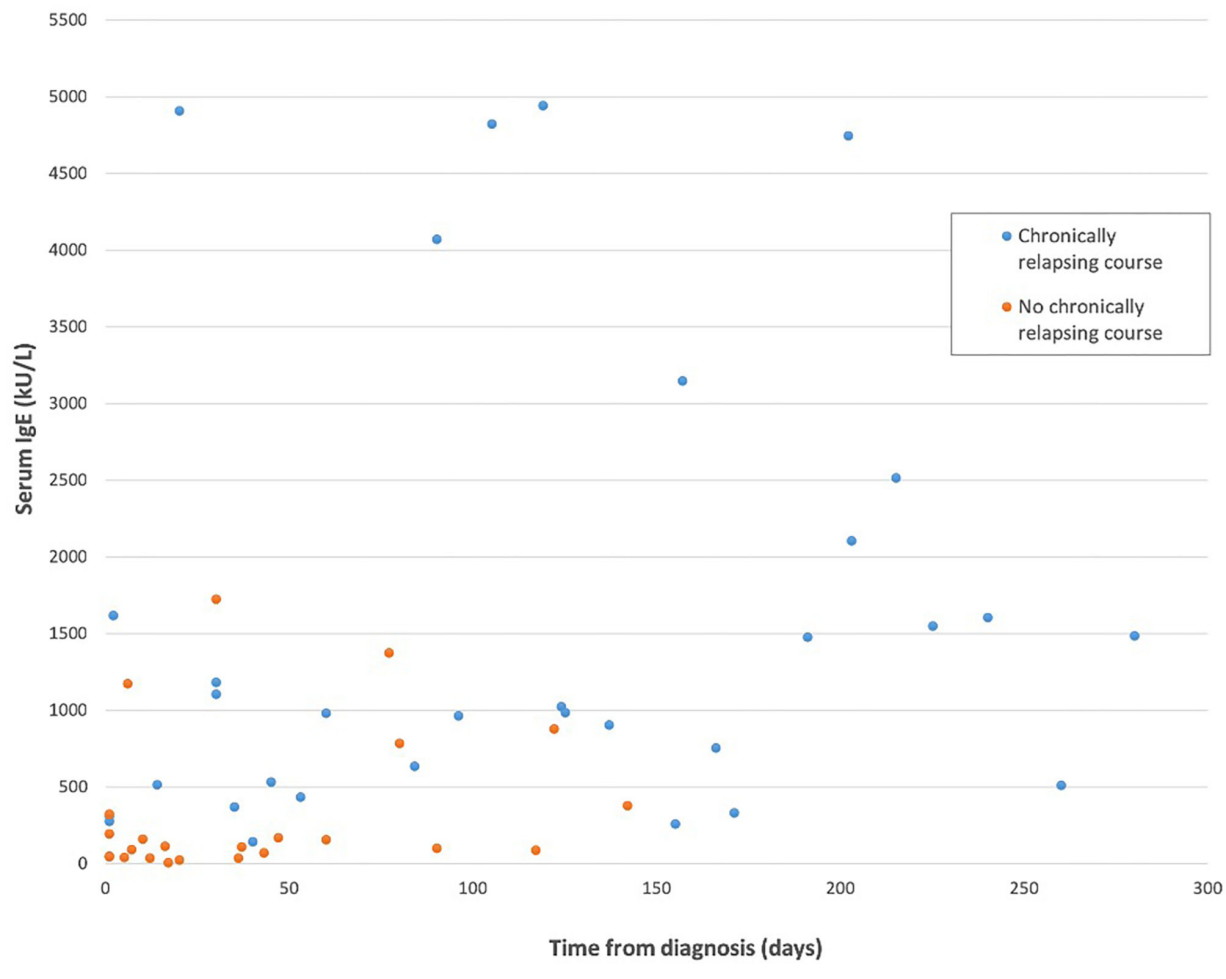
IgE level considering the length of the disease in the two study groups. The patient with hyper-IgE syndrome has been excluded from this figure.

Available IgE levels during the disease course are showed in Figure 1 (*r* = 0.356, *p* = 0.005). IgE levels within individual patients are reported in Supplementary Table 1. IgE level was compared between groups, considering for each patient the first available test. IgE median value was 116 U/mL (IQR: 40–210) in patients without relapses and 1,144.5 U/mL (IQR: 463–2,233.5) in the chronic course group. This difference was statistically significant (*p* < 0.0001). Moreover, the 2 groups were compared considering only the patients for whom IgE value was available within the first month since disease start. Therefore, this analysis was limited to a subgroup of 21/29 patients. IgE level was significantly higher in patients with a successive chronic course with respect to the patients with a shorter course. In particular, median IgE levels were 1,144.5 U/mL (IQR: 361–4,087.5) and 93 U/mL (IQR: 39.5–259), respectively (Table 1B; *p* = 0.003). A very high value of IgE (>10,000 U/mL) was detected in 1 patient with a chronic relapsing course of GCS. The clinical and laboratory scoring system for HyperIgE syndrome (HIES) (13) was applied to all enrolled subjects. Two patients presented a score of >40 that is considered highly likely for HIES and are followed at Immunology Unit. Molecular genetic testing confirmed the pathogenic variant in *STAT 3* HIES in 1 patient. Differences between groups remain statistically significant excluding this child (*p* < 0.0001 considering the first available IgE level; *p* = 0.003 considering IgE tested within the first month).

Personal history of atopy or allergopathy was negative in the study patients, with the exception of 1 child who was positive for specific IgE for food. A complete allergy screening was not performed in the enrolled children, as no children presented anamnestic and clinical criteria suggestive for atopy diathesis (i.e., allergic rhinoconjuntivitis or allergic asthma). No data about family history of atopy was available.

**Figure 2:**
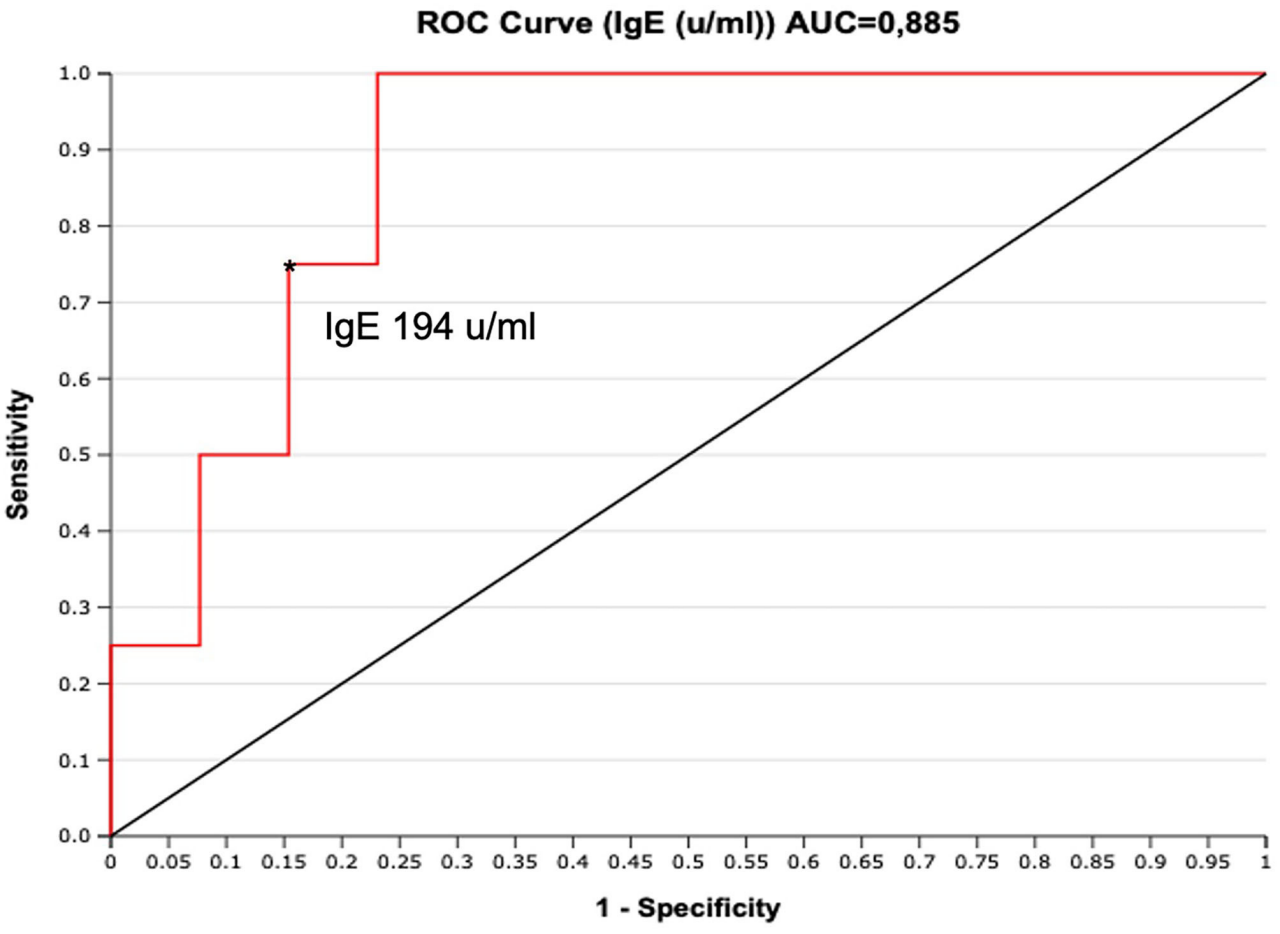
ROC curve for serum IgE (U/ml) performed during the first 30 days from clinical onset. The cut-off of IgE 194 U/ml maximizes the sensitivity (100%, FN = 0), with good specificity (77%, FP = 3). *indicate the cut-off of IgE 194 u/ml.

**Figure 3:**
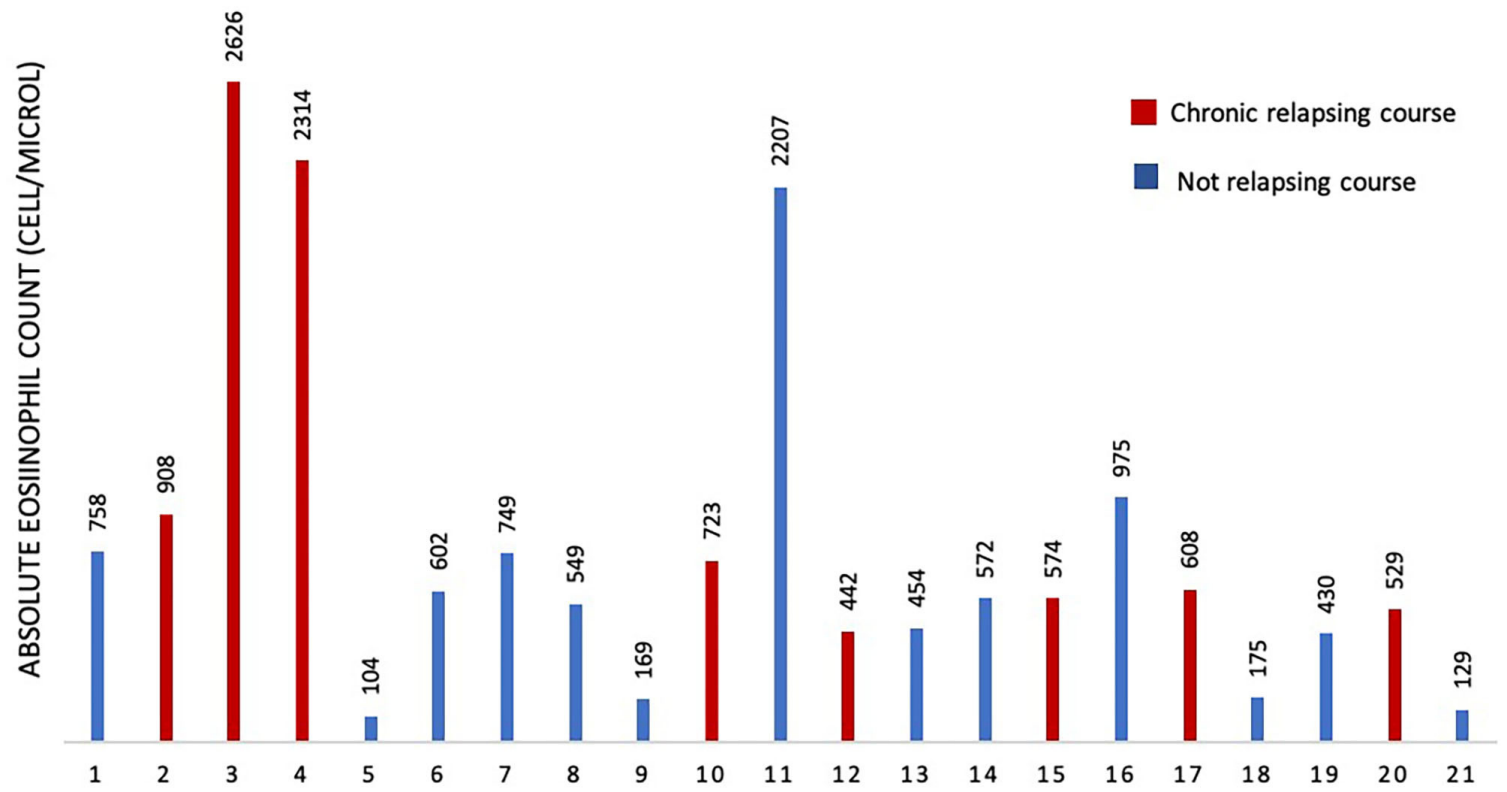
Absolute eosinophil count in 21 patients.

To assess if serum IgE values could be used to preview if GCS patient will develop chronic relapsing course, we designed a ROC curve (Figure 2). We obtained an AUC value that was satisfactory for a diagnostic test (AUC = 0.88). We chose a cut-off value that maximized the percentage of sensitivity. One hundred percentage of GCS patients with chronic relapsing course had serum IgE values superior than 194 U/ml. Therefore, in this study, if a patient affected by GCS during his first 30 days of disease had serum IgE value higher than 194 U/ml he had a high probability (77%, CI 95%: 54–99%) for having a chronic relapsing course. The absolute eosinophil count (AEC) detected within 2 months since the diagnosis of GCS, was available for 21/29 patients (72.4%) (Figure 3). Eosinophilia (>500 cells/μL) was present in 66.6% (14/21) of the children. Median eosinophil count in children without a chronic course was 549 cells/μL (IQR 172–753), whereas in those with a chronic relapsing course was 665.5 cells/μL (IQR 540.25–1,962.5) (*p* = 0.121). In particular, mild eosinophilia (500–1,000 cells/μL) was found in 11/14 patients (78.6%) and 6/11 (54.5%) didn't present a chronic course of GCS. Severe eosinophilia (>1,500 cells/μL) was found in 21.4% (3/14) of the children, and one of them didn't present a chronically relapsing course (as shown in Figure 3).

## Discussion

To the best of our knowledge this is the first study analyzing the correlation of serum IgE level with the clinical course of the GCS. In particular, a significant correlation between higher IgE levels and chronic-relapsing course has been detected in this study. Therefore, IgE may be a predictive factor of the length of the disease. In fact, usually GCS is a self-healing condition, during 2 to 6 weeks, but it may last several weeks with a chronic relapsing course. In view of this, we analyzed 29 patients referred to our hospital from June 2018 to November 2019 with a diagnosis of GCS for whom laboratory tests were available, in particular IgE and, in most cases, complete blood cell count. Of the 29 patients included in the study, we found that about half (51.7%) had a chronically relapsing course. This group of patients had a duration of the disease 5 times longer than the other group. If we compared the median IgE level between the 2 groups, we found a 10-fold IgE value in children with chronic course. Furthermore, we found that this difference of serum IgE value, was already present if the blood tests were done within the first month since onset of the disease.

This results remain significative also excluding a patient with high value of IgE (>10,000) who was confirmed with the pathogenic variant in *STAT 3* of hyper IgE syndrome. A similar case was also described by Chuh (14). Moreover, the ROC curve designed to assess the predicting value of serum IgE values on chronic relapsing course, showed an AUC value that was satisfactory for a diagnostic test. We chose a cut-off value that maximized the percentage of sensitivity. Therefore, in this study, if a patient affected by GCS during his first 30 days of disease had serum IgE value higher than 194 U/ml he had a high probability (77%) for having a chronic relapsing course.

In order to investigate other possible markers of GCS with chronic relapsing course, in this study we analyzed also AEC. This was done considering that in other conditions, such as allergies, IgE levels and AEC are both increased. In fact, as GCS is thought in part to be related to an immunologic response to a viral infection, potentially of hypersensitivity, eosinophilia or high IgE may result. The AEC, detected within 2 months since the diagnosis of GCS, was available for 21/29 patients and no statistical significance was found comparing the 2 groups. This led us to hypothesize that AEC has not a direct correlation with the GCS course. The lack of correlation with blood eosinophilia is not surprising in the light of a histopathological finding of GCS characterized by the presence of an inflammatory infiltrate mainly of lymphocytes and histiocytes with occasional scattered eosinophils (7, 15). Despite this, GCS has been recently described in one case of idiopathic hypereosinophilic syndrome (4).

Considering that no children presented anamnestic and clinical criteria suggestive for atopy diathesis and a personal history of atopy was recorded only in 1 child, we looked at the close connection between the IgE serum level and the clinical course of GCS. The immunopathogenic mechanism of GSC is still obscure. While historically thought to be a manifestation of hepatitis B infection, the association with various viral agents (Coxsackie, CMV, enteroviruses, parvovirus B19, poxvirus, rotavirus, rubella, hepatitis A virus, HSV-1) has been reported and, not rarely, GCS has been described following vaccination (3–6). This suggest a role of an immunologic response to viral antigens rather than a primary manifestation of infection. Our results suggest that, in this immunologic response, IgE may play a role. On the best of our knowledge, only two studies investigated the atopic background in GCS patients, finding a significant association with atopy (8, 9). In the study by Ricci and colleagues, among the 29 patients included, 7 children with GCS (24.1%) presented with atopic dermatitis, according to the criteria of Hanifin and Rajka and five of them had an alteration of IgE values (data not shown in the article) (9). A higher presence of atopic dermatitis and family history for atopy was found in GCS patients compared to controls (24.1 *vs*. 6.8% and 51.7 vs. 31%, respectively) (9). Therefore, the authors conclude that atopy may play an important role in conditioning the onset of GCS in children exposed to different microbiological agents. Moreover, they added that, during the first years of life, atopic subjects may express a “papular prone” phenotype when exposed to different external stimuli, showing papular dermatoses such as frictional lichenoid eruption or lichen striatus, which resembles GCS (15). Likewise, Chuh et al. in their retrospective study, analyzed 37 children with GCS compared to 37 control subjects (8). They found that GCS was significantly associated with atopic dermatitis (28/37), whereas the associations with asthma, allergic rhinitis, atopic urticaria, and allergic conjunctivitis were insignificant (8).

However, in our study a complete allergic screening was not performed for the absence of suggestive clinical symptoms. Differently from those studies we didn't found a correlation with personal history of atopy and with related manifestations, so we cannot confirm the previously reported data. The limited follow-up does not consent to exclude that these children may develop atopic dermatitis or other allergic symptoms in the future, since we followed them in a short time period. Moreover, IgE levels after symptoms resolution were available only for 12/29 patients, and therefore were not analyzed in this study. This was due to the fact that many patients did not come back to visit after clinical recovery. Therefore, it is not possible to assume that there was a complete normalization of IgE levels in both groups and, on the other hand, to define if there was a persistence of this condition.

Otherwise, we focused on the possible role of IgE in the pathogenesis or in the clinical evolution of GCS. The main result of our study is that the higher value of IgE, when detected within the first month to symptoms onset, is a direct predictive factor of the course of GCS. This is important because, even if GCS is a transient and self-healing condition, chronically relapsing course is very common and often associated with itchy symptoms. The knowledge of a possible long-lasting disease supports the physician in reassuring children's parents.

The main limitation is that study group was small and may not be representative of the entire population of children with GCS. Moreover, another limit is the not homogeneous time of blood testing. In fact, despite the prospective design, blood tests were done only in occasion of clinical evaluation. Therefore, IgE levels were not available at the same time for all patients. No microbiological data, including viral serologies, were available, so it was not possible to establish if underlying superinfection could play a role in the chronicity of the lesions. No complete allergological data were available, in particular allergen specific IgE were not done in absence of a personal history of atopy. Moreover, the lack of IgE data after symptoms resolution did not consent to draw conclusions regarding possible differences on the timespan of this alteration. Therefore, we believe that further prospective studies in children could better clarify the role of IgE levels and the immune background of this condition.

## Data Availability Statement

The raw data supporting the conclusions of this article will be made available by the authors, without undue reservation.

## Author Contributions

AB and EV wrote the main manuscript. FP, SR, and TO contributed to the data collection. CF, CA, and LG are the senior supervisors of the work for the dermatologic, immunologic, and pediatric infectious section, respectively. All authors contributed to the article and approved the submitted version.

## Conflict of Interest

The authors declare that the research was conducted in the absence of any commercial or financial relationships that could be construed as a potential conflict of interest.
