# Supplementary material for: The Serum Immunoglobulin E Level: Is There a Relationship With the Clinical Course of the Gianotti-Crosti Syndrome?
Source: Front Pediatr. 2021 Feb 25;9:643341. doi: 10.3389/fped.2021.643341 (PMC7947791; doi:10.3389/fped.2021.643341)
Supplement: Supplementary file 1 [file Table_1.DOCX]

| Study group | Age (months), gender | IgE 0-30 days  (U/mL) | IgE 31-90 days  (U/mL) | IgE 91-180 days  (U/mL) | IgE >181 days  (U/mL) |
| --- | --- | --- | --- | --- | --- |
| Non chronically relapsing course | 33, F | 324 |  | 87 |  |
|  | 27, M | 93 |  |  |  |
|  | 31, M | 41 |  |  |  |
|  | 16, F |  | 785 | 377 |  |
|  | 24, F | 162 | 170 |  |  |
|  | 12, F | 50 | 35 |  |  |
|  | 23, F | 116 | 108 |  |  |
|  | 14, M | 45 | 103 |  |  |
|  | 41, F | 194 | 70 |  |  |
|  | 30, M | 38 |  |  |  |
|  | 12, F | 1174 |  | 878 |  |
|  | 15, M | 1724 | 1375 |  |  |
|  | 15, F | 26 |  |  |  |
|  | 26, F | 5 |  |  |  |
|  | 9, F |  | 157 |  |  |
| Chronically relapsing course | 34, F |  | 4074 | 3148* | 1030 |
|  | 16, M§ | 12180 |  | 10560 |  |
|  | 27, F | 1620 |  | 965 |  |
|  | 12, M |  | 143 | 334 | 2515* |
|  | 12, M | 4910 | 7090* | 4945 | 4747 |
|  | 13, M | 514 | 371 | 903* |  |
|  | 24, M |  |  |  | 1550* |
|  | 7, F | 276 | 436* |  |  |
|  | 15, M | 310 | 531 |  | 2105* |
|  | 28, M |  |  |  | 1605* |
|  | 36, M |  |  | 986 | 1478* |
|  | 20, M |  | 980* | 259 | 510* |
|  | 32, F | 1107 |  | 4824 |  |
|  | 13, F | 1182 |  | 1025* |  |

**Supplementary table 1.** IgE levels for each patients during the study period.

§ Patient with Iper-IgE syndrome

* IgE level in course of Gianotti-Crosti relapse
